# Driving Naive State Induction Using Human Wharton Jelly-Mesenchymal Stem Cell-Derived Conditioned Medium in Rhesus Monkey Embryonic Stem Cells

**DOI:** 10.3390/cells15070626

**Published:** 2026-03-31

**Authors:** Preeyanan Anwised, Ratree Moorawong, Worawalan Samruan, Jittanun Srisutush, Sirilak Somredngan, Irene Aksoy, Pierre Savatier, Rangsun Parnpai

**Affiliations:** 1 Embryo Technology and Stem Cell Research Center, School of Biotechnology, Institute of Agricultural Technology, Suranaree University of Technology, Nakhon Ratchasima 30000, Thailand; preeyanan.a@sut.ac.th (P.A.); ratreem1998@gmail.com (R.M.); worawalan.sa@sut.ac.th (W.S.); jittanunsrisutush@gmail.com (J.S.); 2 Medeze Group Public Company Limited, Nakhon Pathom 73220, Thailand; sirilak@medezegroup.com; 3 Univ Lyon, Université Lyon 1, INSERM, Stem Cell and Brain Research Institute U1208, 69500 Lyon, France; irene.aksoy@inserm.fr; 4 PrimaStem Platform, Univ Lyon, Université Lyon 1, INSERM, Stem Cell and Brain Research Institute U1208, 69500 Lyon, France

**Keywords:** conditioned medium, embryonic stem cells, mesenchymal stem cells, primed state, naive state, rhesus monkey

## Abstract

**Highlights:**

**What are the main findings?**

- hWJ-MSCs promote primed-to-naive conversion in RhESCs.
- hWJ-MSCs-CM more robustly induced the expression of key naive markers, including *KLF4*, *KLF17*, *ESRRB*, *TFAP2C* and *DPPA2*.

**What is the implication of the main finding?**

- hWJ-MSCs-CM as a novel culture medium for enhancing naive pluripotency in primate ESCs.
- hWJ-MSCs provide a human-compatible, xeno-free alternative to traditional MEFs-based systems.

**Abstract:**

The conversion of primed pluripotent stem cells to a naive-like state has emerged as a critical strategy for enhancing developmental potential and broadening applications in regenerative medicine. Conditioned media (CM)-based approaches provide a supportive microenvironment enriched with secreted factors that may facilitate this state transition without extensive genetic or chemical manipulation. In this study, we investigated the potential of human Wharton’s Jelly-derived mesenchymal stem cell-conditioned media (hWJ-MSCs-CM) and mouse embryonic fibroblasts CM (MEFs-CM) to support the conversion of primed rhesus monkey embryonic stem cells (rhESCs) into a naive-like state. The rhESCs were cultured under feeder-free and feeder conditions using both hWJ-MSCs-CM and MEFs-CM, exhibiting distinct morphological changes during conversion. Immunofluorescence analysis demonstrated the expression of pluripotency and naive markers under both conditions. Gene expression analysis further confirmed the upregulation of naive-specific genes and downregulation of primed markers, with statistically significant differences between groups. Additionally, epigenetic reprogramming was assessed, revealing differential effects of the CM sources on the reversion to a naive state. These findings highlight the potential of hWJ-MSCs-CM as a supportive system for naive-like state induction in primate ESCs.

## 1. Introduction

In rodents, pluripotent stem cells (PSCs) occupy two functionally distinct states of pluripotency: a naive state exemplified by embryonic stem cells (ESCs) and a primed state represented by epiblast stem cell lines (EpiSCs) [1]. These two states exhibit profound differences in their transcriptional and epigenetic landscapes, which critically shape their biological characteristics and functional properties [2]. Historically, human PSCs have existed in a primed-like state, reliant on FGF2 and activin A to suppress differentiation. Only a few naive ESC lines have been successfully derived from human blastocysts [3]. Achieving the naive state typically involves culturing in defined cocktails containing growth factors and small-molecule inhibitors (e.g., PKC, MEK, and GSK3β inhibitors). However, the resulting cell lines are often unstable. A more reproducible strategy involves resetting primed ESCs or iPSCs using reprogramming factors that restore naive-like properties [4,5,6]. Naive PSCs also exhibit several functional advantages: they tolerate single cell dissociation and proliferate more rapidly than their primed counterparts. Importantly, they also tend to be more genetically stable, enhancing their utility in genome editing and biomedical applications [2]. However, the efficiency of naive conversion is highly dependent on culture conditions. Non-human primate (NHPs) ESCs and iPSCs have required more tailored approaches for naive conversion. Fang et al. (2014) pioneered this in rhesus monkeys using 4i/L/b medium supplemented with LIF, FGF2, and inhibitors targeting MEK, GSK3β, p38MAPK, and JNK, which gradually induced the formation of dome-shaped colonies characteristic of naive PSCs [7]. Other studies reported naive state reprogramming using increasingly complex cocktails of small molecules: the NHSM/NHSMV cocktail in cynomolgus ESCs [8], and the multi-condition NHSMV/2iLD/K3cLD/K5cLD approach [9]. Bergmann et al. (2022) developed PLAXA medium to reprogram marmoset ESCs (CjESCs) to naive-like pluripotency using an FGF2/KSR base enriched with MEK and WNT inhibitors, LIF, activin A, and ascorbic acid [10]. Notably, the 4CL protocol [11], which efficiently converted primed CyESCs to a naive state with LIF, activin A, and inhibitors of MEK, tankyrases, S-adenosyl homocysteine hydrolase (SAHH), and histone deacetylases (HDACs), yielding KLF17-enriched, chromosomally stable cells. We recently developed a new culture medium, named VALGöX, for converting primed rabbit and NHP iPSCs to the naive state [12]. This medium consists of fibroblast-conditioned N2B27 supplemented with vitamin C, LIF, activin A, the PKC inhibitor Gö6983, and the tankyrase inhibitor XAV939.

Over the past two decades, there has been a growing interest in the development and characterization of conditioned media (CM), particularly in the context of regenerative medicine, where CM is increasingly viewed as a promising cell-free therapeutic product [13]. Most CM used in PSCs culture are derived from mouse embryonic fibroblasts (MEFs). On the other hand, mesenchymal stem cells (MSCs), found in various tissues, are multipotent and can differentiate into adipocytes, chondrocytes, and osteocytes [14,15,16]. Their therapeutic relevance is largely attributed to their immunomodulatory capacity and the secretion of cytokines and growth factors involved in tissue repair and inflammation regulation [15,16]. While CM from MEFs remains widely used, human Wharton’s Jelly-derived mesenchymal stem cells-conditioned media (hWJ-MSCs-CM) have emerged as a promising alternative, offering similar support for pluripotent stem cell cultures with lower xenogeneic risks. More recently, hWJ-MSCs-CM has attracted attention as a potential cell-free agent for protective and regenerative therapies [17]. In this study, we evaluated the effectiveness of two different sources of conditioned media—MEFs-CM and hWJ-MSCs-CM promoting the conversion of primed rhesus ESCs (rhESCs) to the naive state. Through morphological assessments, immunofluorescence staining, and gene expression analyses, we compared the capacity of each CM to induce naive pluripotency features. This work aims to provide new insights for optimizing naive culture systems in non-human primate ESCs.

## 2. Materials and Methods

### 2.1. Chemicals and Reagents

Unless stated otherwise, all chemicals and reagents were purchased from Sigma-Aldrich (St. Louis, MO, USA). The cell culture media were purchased from Gibco (Paisley, UK), and plastic cell culture devices were obtained from SPL Life Sciences (Pocheon-si, Republic of Korea).

### 2.2. Cells and Culture Media

The Rzh11ESC cell line [18] was obtained from the Yerkes National Primate Research Center (Atlanta, GA, USA). Primed cells were routinely maintained in knockout Dulbecco’s modified Eagle’s medium (KO-DMEM, Gibco) supplemented with 20% knockout serum replacement (KOSR, Gibco), 1 mM glutamine, 0.1 mM β-mercaptoethanol (Sigma-Aldrich), 1% non-essential amino acids (NEAA, Gibco), and 4 ng/mL FGF2 (Gibco). Cultures were grown on mitotically inactivated mouse embryonic fibroblasts (MEFs) at a density of 2.5 × 10^5^ cells per 35 mm dish. Media were changed daily, and cells were passaged every 3–4 days via mechanical dissociation. Mouse embryonic fibroblasts (MEFs) were isolated from 12.5-day-old mouse embryos (Charles River, Wilmington, MA, USA) following the protocol by Afanassieff et al. [19]. Briefly, embryos were dissected, decapitated, and eviscerated. Embryonic tissues were minced and incubated with 5× trypsin for 10 min at 37 °C. The digested tissue was centrifuged at 450× *g* for 10 min, and the cell pellet was resuspended in fibroblast medium (DMEM supplemented with 10% FBS, 1× NEAA, 1% PSG) and plated onto 100 mm dishes. After 2–3 days of culture, MEFs were expanded and cryopreserved at 2 × 10^6^ cells/vial using CryoStor^®^ CS10 medium (Stem Cell Technologies, Vancouver, BC, Canada). Human Wharton’s Jelly-derived mesenchymal stem cells (hWJ-MSCs) were obtained via material transfer agreement [20], under ethical approval EC-61-58 (Suranaree University of Technology). Cells were cultured in α-MEM supplemented with 2 mM L-glutamine, 100 U/mL penicillin, 100 µg/mL streptomycin, and 10% fetal bovine serum (FBS, Gibco).

### 2.3. hWJ-MSCs Characterization

Colony-forming unit (CFU) assay: a total of 200 cells of hWJ-MSCs were seeded per well in 6-well plates and cultured for two weeks with media changes every 2–3 days. Cells were fixed with 4% paraformaldehyde (PFA) for 20 min, stained with 0.5% crystal violet, and examined microscopically (Eclipse Ti-S, Nikon, Tokyo, Japan). Colonies (≥50 cells) were manually counted. CFU efficiency was calculated as:

% CFU = (Number of colonies × 100)/Initial number of seeded cells.

Population doubling time (PDT) assay: cells at passages 4–10 was seeded at 4000 cells/cm^2^ in 35 mm dishes and cultured for 72 h. Viable cells were counted using 0.4% trypan blue.

PDT was calculated as: PDT = (t × log2)/(log NF − log NI)

where t = time in hours, NI = initial cell number, NF = final cell number.

Flow cytometry analysis: hWJ-MSCs (passage 5) were labeled with the following antibodies: CD73-APC, CD90-APC/A750, CD105-PE (Biolegend, San Diego, CA, USA, 1:100), CD34-PE (Beckman Coulter, Brea, CA, USA, 1:10), and CD45-FITC (Biolegend, 1:20). Isotype controls were used. After a 20 min incubation in the dark, cells were washed with PBS and analyzed using an Attune™ NxT Flow Cytometer (Thermo Fisher Scientific, Waltham, MA, USA). Differentiation assays: cells at passage 5 were cultured on 0.1% gelatin-coated 6-well plates until 80% confluence and induced with lineage-specific media. Osteogenic medium consisted of α-MEM with 100 nM dexamethasone, 0.2 mM ascorbate-2-phosphate, 10 mM β-glycerophosphate; adipogenic medium consisted of α-MEM with 10 µM insulin, 100 µM indomethacin, 1 µM dexamethasone, 0.5 mM IBMX (IBMX removed after 7 days) and chondrogenic medium consisted of α-MEM with 10 µg/mL ITS-X, 50 µg/mL ascorbate-2-phosphate, 40 µg/mL proline, 100 µg/mL sodium pyruvate, 100 nM dexamethasone, 10 ng/mL TGF-β3, and 2% FBS. Media were changed every 3 days over 21 days. Staining included alizarin red (osteogenesis), oil red O (adipogenesis), and alcian blue 8x (chondrogenesis). Cells were visualized by inverted microscopy (Eclipse Ti-S, Nikon Imaging Japan Inc., Tokyo, Japan) using the NIS-Elements D program (Nikon Imaging Japan Inc.).

### 2.4. Conditioned Media Preparation

Cells (MEFs at 4 × 10^6^ and hWJ-MSCs at 3 × 10^6^) at passages 4 and 7, respectively, were treated with 5 µg/mL mitomycin C. Following treatment, the cells were seeded into 10 cm culture dishes and allowed to reach 80–90% confluence in standard culture medium over a period of 24 h. After this incubation, the medium was replaced with 25 mL of N2B27 basal medium, as previously described [21], supplemented with 20 ng/mL bFGF, and the cells were cultured for an additional 72 h. Conditioned media (CM) were collected daily, filtered through a 0.2 µm filter, and stored at −20 °C until further use. The conditioned media derived from MEFs and hWJ-MSCs are hereafter referred to as MEFs-CM and hWJ-MSCs-CM, respectively.

### 2.5. Conversion of rhESCs to the Naive State

Primed colonies were dissociated into single cells using 1 × TrypLE™ and subsequently seeded onto two different feeder cell types: MEFs (2.8 × 10^5^ cells per 35 mm dish) and hWJ-MSCs (5.0 × 10^5^ cells per 35 mm dish). The cells were cultured in a mixed medium referred to as VALGöX, as established by Pham et al. [12], consisting of CMs from both MEFs-CM and hWJ-MSCs-CM, supplemented with 250 µM ascorbic acid, 10 ng/mL activin A (Peprotech, Cranbury, NJ, USA), in-house-produced LIF, 1.25 µM Gö6983 (Bio-Techne, Minneapolis, MN, USA), and 2.5 µM XAV939 (Sigma-Aldrich). Cells were maintained at 37 °C in a humidified atmosphere containing 5% CO_2_ and 5% O_2_. The medium was replaced daily, and cells were passaged every three days. On Day 9, the culture medium was switched to ALGöX (identical to VALGöX but without ascorbic acid), and the cells were cultured under this condition until Day 21. For feeder-free culture, cells were seeded onto culture dishes pre-coated with 5 µg/mL laminin-521 (Biolamina, Sundbyberg, Sweden) using the same protocol.

### 2.6. Immunofluorescence and Imaging

Cells were seeded on 15 mm glass coverslips (Thermo Fisher Scientific) before the staining. They were fixed with 4% PFA for 20 min at room temperature (RT), washed three times for 5 min in PBS at RT and permeabilized in 0.5% Triton X-100 for 30 min. After three washes in PBS, cells were kept in blocking solution (2% bovine serum albumin) for 1 h at RT [22]. The cells were then incubated overnight in primary antibodies at 4 °C. The following day, cells were washed in PBS and incubated in secondary antibodies for 1 h at RT. The list of antibodies used in this study is presented in Table 1. Cells were then washed three times in PBS prior to mounting in Prolong antifade mounting medium with DAPI (Vector Laboratories, Burlingame, CA, USA) and visualized using a fluorescence inverted microscope (Eclipse TE 300, Nikon Imaging Japan Inc.) with NIS-Elements D program (Nikon Imaging Japan Inc., Tokyo, Japan). The expression of protein markers was quantified by analyzing fluorescence intensity using the FIJI software version 2.16.0. The immunofluorescence quantifications were analyzed using total cell counts (DAPI) from two independent biological replicates.

### 2.7. Real-Time Quantitative PCR (qPCR)

Total RNA was extracted using FavorPrep Tissue Total RNA Mini Kit (Favorgen, Ping Tung Biotechnology Park, Ping-Tung, Taiwan). cDNA synthesis was performed using cDNA Synthesis Kit (Biotechrabbit, Berlin, Germany) and qRT-PCR was performed using KAPA SYBR^®^ FAST qPCR Master Mix on a QuantStudio™ 5 system. Cycling parameters were 95 °C for 3 min, followed by 40 cycles of 95 °C for 15 s, 60 °C for 30 s, and 72 °C for 30 s, with a final extension at 72 °C for 5 min. Melting curve analysis confirmed primer specificity (Table 2). Gene expression was quantified using the 2^−ΔΔCt^ method and normalized to GAPDH.

### 2.8. Statistical Analysis

All statistical analyses were conducted using GraphPad Prism version 5 (GraphPad Soft-ware, San Diego, CA, USA). Data are expressed as the mean ± standard deviation (SD). Group differences were evaluated by one-way analysis of variance (ANOVA), followed by Tukey–Kramer’s honestly significant difference (HSD) post hoc test for multiple pair-wise comparisons. Statistical significance was defined as a *p*-value < 0.05. Levels of significance are denoted as follows: * *p* < 0.05, ** *p* < 0.01, and *** *p* < 0.001.

## 3. Results

### 3.1. hWJ-MSC Characterization

**Figure 1:**
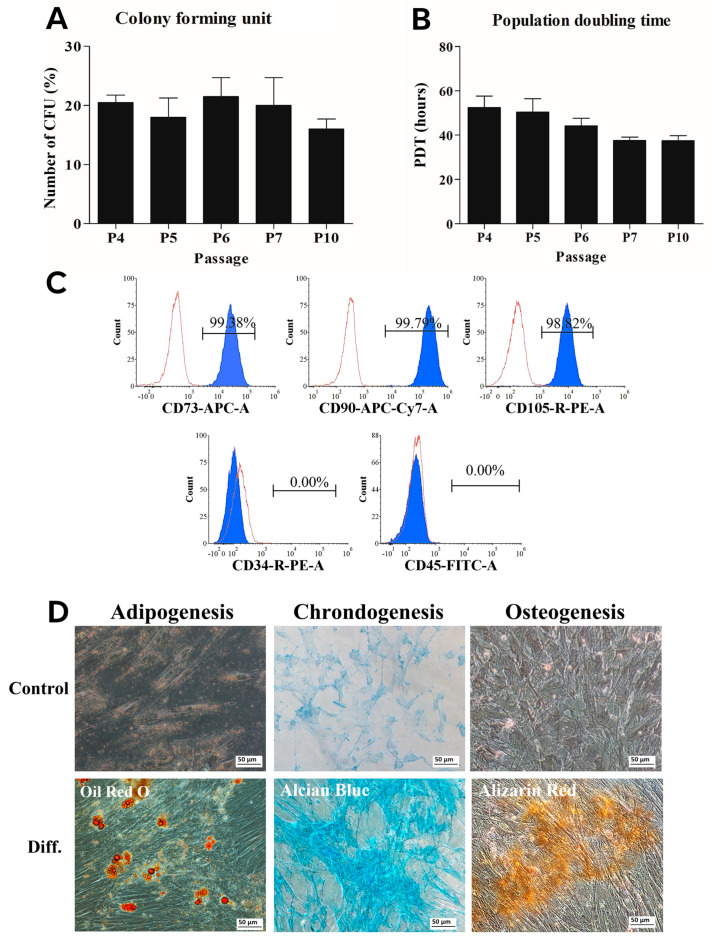
Characterization of hWJ-MSCs. (**A**) Colony-forming unit assay. (**B**) Population doubling time. (**C**) Cell surface expression analyzed by flow cytometry using CD73^+^, CD90^+^, CD105^+^, CD34^−^, and CD45^−^ antibodies. (**D**) Tri-lineage differentiation potential of hWJ-MSCs after 21 days, assessed by oil red O (adipogenesis), alcian blue (chrondogenesis), and alizarin red (osteogenesis). Scale bar, 50 μm.

The characteristics of hWJ-MSCs were assessed using standard procedures [20]. The colony-forming unit (CFU) assays conducted at passage 4, 5, 6, 7 and 10 yielded values ranging from 16 ± 1.7 to 21.5 ± 3.2 (Figure 1A). The population doubling times (PDts) at passages (P) 4 to 7 and 10 ranged from 38.5 ± 2.2 h to 52.5 ± 5.2 h (Figure 1B), values consistent with those previously reported for hWJ-MSCs derived from human umbilical cord Wharton’s jelly [23]. Flow cytometry confirmed that hWJ-MSCs were positive for CD73, CD90, and CD105, and negative for CD34 and CD45 (Figure 1C), with over 95% of cells expressing the positive markers and fewer than 2% expressing the negative markers. Furthermore, hWJ-MSCs demonstrated tri-lineage differentiation potential. Adipogenic differentiation was evidenced by the formation of lipid droplets after 21 days, chondrogenic differentiation by the production of glycosaminoglycan-rich extracellular matrix, and osteogenic differentiation by the appearance of calcium deposits–evidenced using alizarin red staining (Figure 1D). All these results are consistent with the criteria set by the International Society for Cell and Gene Therapy [24,25,26].

### 3.2. Propagation of Rzh11ESCs Using MEFs and hWJ-MSCs-Derived Feeder Cells

**Figure 2:**
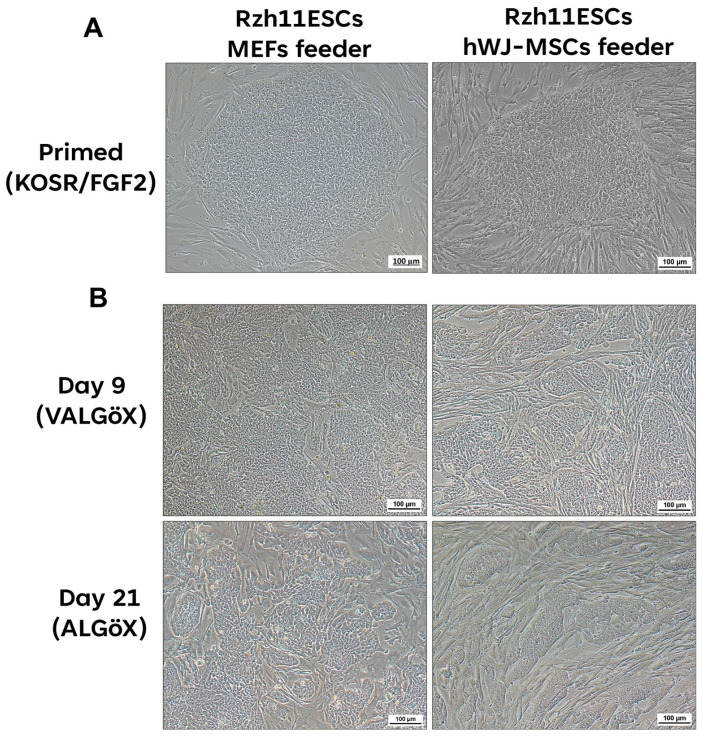
Morphology of Rzh11ESCs cultured under primed conditions and following conversion to the naive state. (**A**) Propagation of primed Rzh11ESCs on MEFs and hWJ-MSCs-derived feeder cells in KOSR/FGF2 medium. (**B**) Morphological changes observed for 21 days conversion of Rzh11ESCs in VALGöX/ALGöX medium on feeder cells. Scale bars: 100 μm.

**Figure 3:**
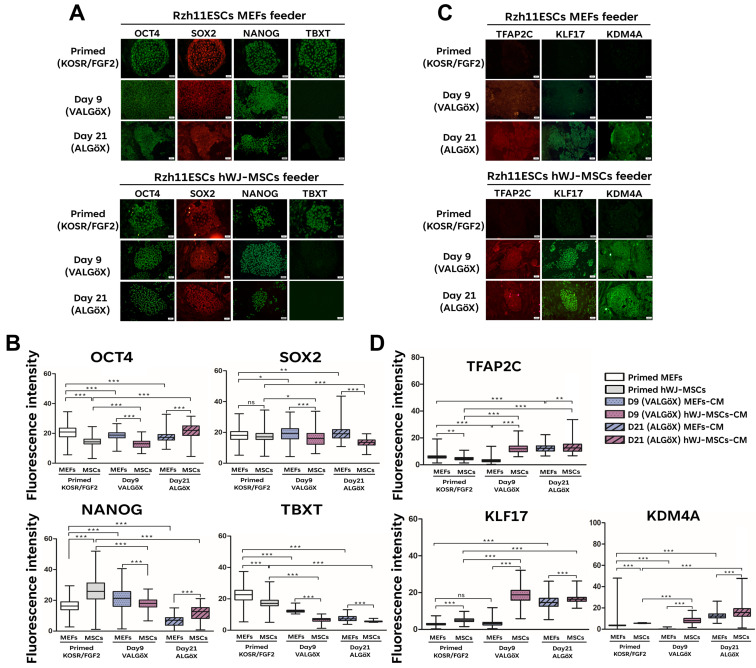
Reversion of Rzh11ESCs from the primed to the naive-like state using MEFs and hWJ-MSCs feeder cells. (**A**) Expression of pluripotency markers during the 21-day conversion on feeder cells. (**B**) Quantification of pluripotency markers intensities over 21 days. (**C**) Expression of naive markers during the 21-day conversion on feeder cells. (**D**) Quantification of naive markers intensities over 21 days. Scale bars: 50 μm Statistical significance was determined by one-way ANOVA followed by Tukey’s multiple comparison test. Comparisons were made across groups: * *p* < 0.05, ** *p* < 0.01, *** *p* < 0.001, ns: not significant.

**Figure 4:**
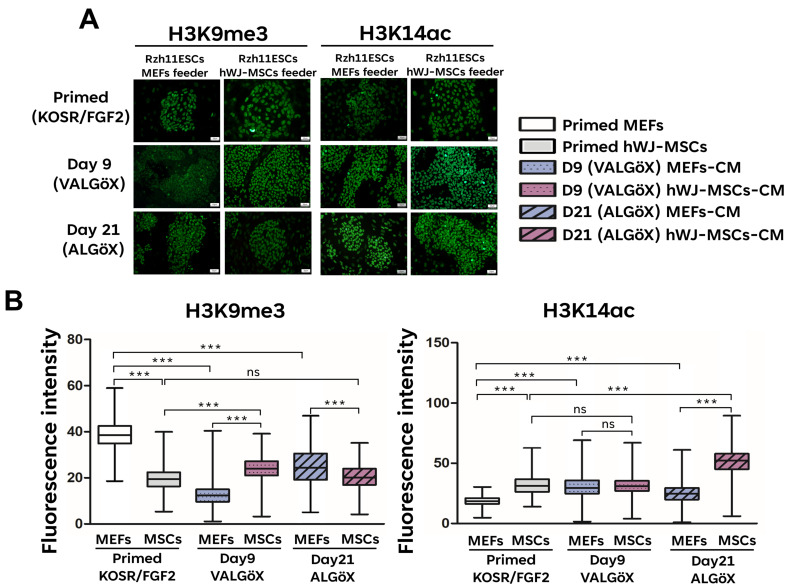
Epigenetic changes in Rzh11ESCs during the transition from primed to naive-like state using MEFs and hWJ-MSCs feeder cells (**A**) Representative images showing epigenetic changes during the primed-to-naive transition. (**B**) Quantification of epigenetic marker intensities over 21 days. Scale bars: 50 μm. Statistical significance was determined using one-way ANOVA followed by Tukey’s multiple comparison test. Comparisons were made across groups: *** *p* < 0.001, ns: not significant.

The rhesus ESC line Rzh11 (Rzh11ESCs) was originally derived using growth-inactivated MEFs as feeder cells in the presence FGF2 and KOSR, resulting in self-renewal in the primed pluripotent state [18]. In the present study, we found that Rzh11ESCs could also be maintained on feeders derived from hWJ-MSCs. These cells formed flat colonies typical of the primed pluripotency state, demonstrating that hWJ-MSCs-derived feeder cells can support the self-renewal of Rzh11ESCs (Figure 2A). To assess whether hWJ-MSCs-derivedfeeders could also support self-renewal of Rzh11ESCs in a naive-like pluripotent state, we cultured the cells in VALGöX–a medium recently shown to facilitate the conversion of primed rabbit iPSCs to a naive-like state [12]. Rzh11ESCs were cultured in VALGöX for 9 days on either growth-inactivated MEFs or hWJ-MSCs, followed by 12 days in ALGöX, a modified formulation lacking vitamin C. Under both feeder conditions, Rzh11ESCs underwent morphological changes indicative of the transition from a flattened, epithelial-like morphology to more compact colonies. Notably, cells cultured on hWJ-MSCs-derived (Day 21) feeders exhibited more pronounced colony condensation and tighter intercellular junctions compared to those on MEFs-derived feeders, suggesting a potentially more rapid induction of naive characteristics (Figure 2B). By Day 21, Rzh11ESCs on hWJ-MSCs-derived feeders formed well-defined, dome-shaped colonies with smooth borders—morphological characteristics of the naive pluripotent state. In contrast, cells cultured on MEFs exhibited partial reprogramming, forming a mixture of flattened and semi-compact colonies, indicative of heterogeneous or incomplete naive conversion. To further assess the pluripotency status of Rzh11ESCs propagated on MEFs- and hWJ-MSCs-derived feeders, we analyzed the expression of key markers by immunofluorescence. The expression levels of the core pluripotency markers OCT4, SOX2, and NANOG remained comparable between cells maintained in the primed state and those cultured in VALGöX/ALGöX conditions on either feeder type. (Figure 3A,B). However, Rzh11ESCs cultured in VALGöX/ALGöX on both feeders showed upregulated expression of naive pluripotency markers TFAP2C, KDM4A, and KLF17, along with downregulation of the primed marker TBXT (Figure 3C,D). Additionally, we observed epigenetic changes consistent with the primed-to-naive transition, including reduced levels of H3K9me3 level and increased levels of H3K14ac at Days 9 and 21 (Figure 4A,B). Together, these results demonstrate that hWJ-MSCs-derived feeder cells not only support the self-renewal of Rzh11ESCs in the primed state but also facilitate their conversion to a naive-like state, as evidenced by morphological and epigenetic changes.

### 3.3. Propagation of Rzh11ESCs Using hWJ-MSCs-Derived Conditioned Medium

**Figure 5:**
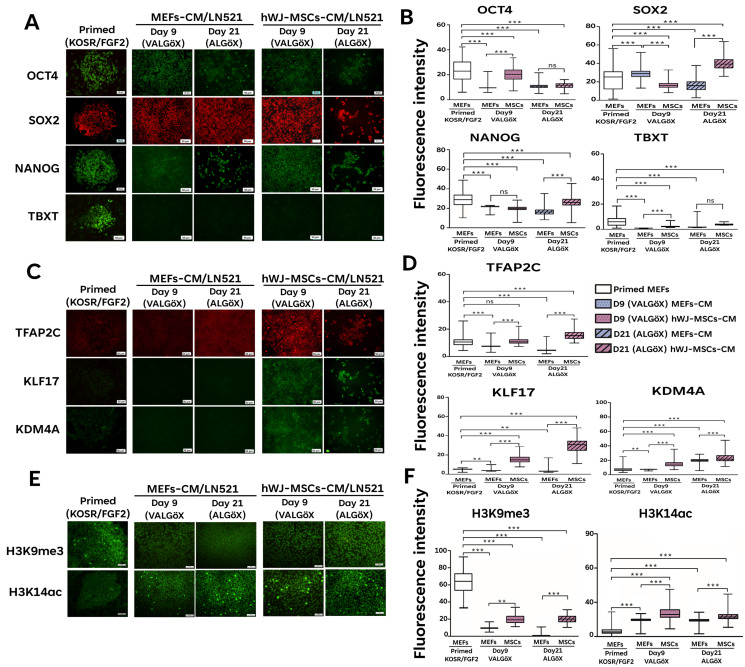
Feeder-free reversion of primed Rzh11ESCs to the naive state using hWJ-MSCs-conditioned medium. (**A**) Expression of pluripotency markers during 21-day conversion on laminin-521. (**B**) Quantification of pluripotency markers intensities over 21 days. (**C**) Expression of naive markers during 21-day conversion on laminin-521. (**D**) Quantification of pluripotency markers intensities over 21 days. (**E**) Epigenetic changes during the primed-to-naive transition. (**F**) Quantification of epigenetic marker intensities over 21 days on laminin-521. Scale bars, 50 μm. Statistical significance was determined using one-way ANOVA followed by Tukey’s multiple comparison test. Comparisons were made across groups: ** *p* < 0.01, *** *p* < 0.001, ns: not significant.

We investigated the capacity of conditioned medium (CM) derived from hWJ-MSCs to support the self-renewal of Rzh11ESCs under feeder-free culture conditions. To this end, Rzh11ESCs initially cultured on MEFs in KOSR/FGF2 were transferred to laminin-521-coated dishes and cultured in VALGöX medium supplemented with either MEFs-CM or hWJ-MSCs-CM for 9 days, followed by 12 days in ALGöX. Under both conditions, Rzh11ESCs maintained an undifferentiated morphology, with no discernable differences in overall appearance. However, significant differences were observed in the pluripotency-associated genes. Immunofluorescence analysis revealed variable expressions of the core pluripotency factors OCT4, NANOG, and SOX2 in Rzh11ESCs transitioned from the primed to the naive-like state under feeder-free condition (Figure 5A,B). Notably, cell cultured in hWJ-MSCs-CM exhibited significantly higher fluorescence intensities. Naive-specific markers TFAP2C, KDM4A, and KLF17 were upregulated in cells cultured with hWJ-MSCs-CM at both time points, with KDM4A and KLF17 undetectable in the primed state (Figure 5C,D). Conversely, TBXT expression was markedly downregulated under both CM conditions, further supporting the acquisition of a naive-like state. Finally, immunofluorescence analysis showed a substantial reduction in H3K9me3 levels in both reversion conditions compared to the primed control, accompanied by a strong increase in H3K14ac expression in converted cells (Figure 5E,F). These findings highlight epigenetic reconfiguration marked by a loss of repressive histone methylation and a gain of permissive histone acetylation. Collectively, these findings underscore the ability of hWJ-MSCs-CM promote the self-renewal of Rzh11ESCs in a naive-like state.

### 3.4. hWJ-MSCs-Derived Conditioned Medium Enhances Primed-to-Naive State Conversion

**Figure 6:**
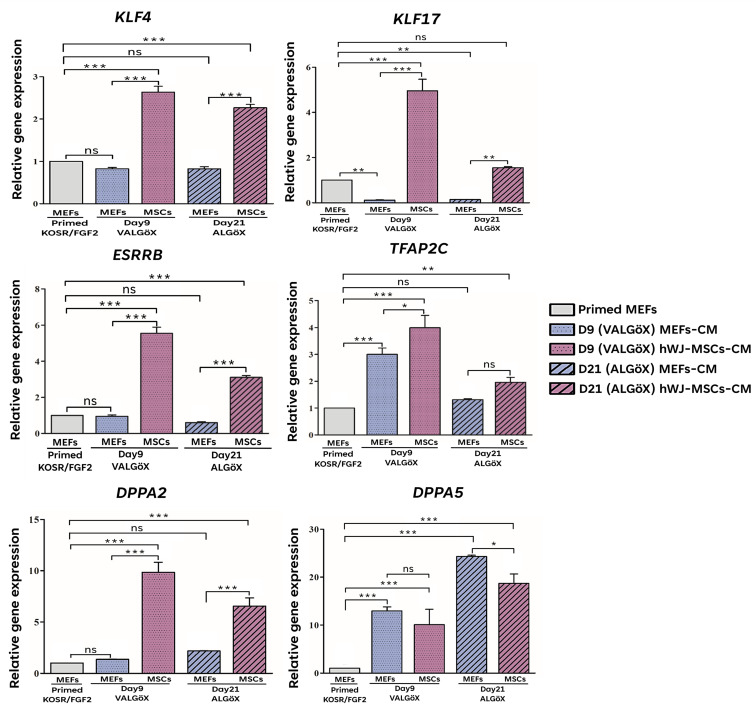
Enhanced primed-to-naive conversion of Rzh11ESCs by hWJ-MSCs-conditioned medium. Expression levels of naive markers (*KLF4*, *KLF17*, *ESRRB*, *TFAP2C*, *DPPA2*, *DPPA5*) in Rzh11ESCs cultured with MEFs-CM or hWJ-MSCs-CM. Gene expression was normalized to GAPDH, and relative expression levels were calculated for each group. Data are presented as mean ± S.D. Statistical significance was determined by one-way ANOVA followed by Tukey’s multiple comparison test. Comparisons were made across groups: * *p* < 0.05, ** *p* < 0.01, *** *p* < 0.001, ns: not significant.

We observed that Rzh11ESCs cultured in ALGöX supplemented with hWJ-MSCs-derived CM exhibited higher expression levels of the pluripotency markers TFAP2C, KDM4A, and KLF17 compared to those cultured in MEFs-CM (Figure 5C,D). This suggests that hWJ-MSCs-CM is more effective in supporting the naive pluripotent state. To examine this further, we analyzed the expression of naive pluripotency markers in Rzh11ESCs cultured in ALGöX with either hWJ-MSCs-CM or MEFs-CM using qPCR. The naive-associated genes *KLF4*, *KLF17*, *ESRRB*, *TFAP2C*, *DPPA2*, and *DPPA5* were significantly upregulated in both conditions (Figure 6). Notably, cells reprogrammed in hWJ-MSCs-CM exhibited significantly higher expression levels of *KLF4*, *KLF17, ESRRB*, *TFAP2C*, and *DPPA2* compared to those reprogrammed in MEFs-CM, indicating a more robust induction of the naive transcriptional program.

## 4. Discussion

Previous studies have shown that MEFs-based feeder systems and their conditioned media can effectively support the reversion of primed human ESCs to a naive-like state, typically characterized by the upregulation of naive state-specific transcription factors, including KLF4, KLF17, ESRRB, TFAP2C, DPPA2, and DPPA5, increased acetylation of H3K4 and H3K14, and decreased methylation of K3K9 and H3K27 [27,28]. In our study, we investigated whether feeder cells and conditioned media derived from human Wharton’s Jelly-mesenchymal stem cells (hWJ-MSCs) could similarly support naive induction in rhesus ESCs (Rzh11ESCs). Our findings demonstrate that hWJ-MSCs provide a supportive environment for both the maintenance of primed pluripotency and the induction of a naive-like state, particularly under chemically defined, feeder-free conditions. Interestingly, the expression of naive markers such as *KLF4*, *KLF17*, and *DPPA2* was already elevated in Rzh11ESCs cultured on hWJ-MSCs-derived feeders under standard primed conditions. This suggests that hWJ-MSCs may secrete bioactive factors that activate components of the naive transcriptional network, even in the absence of naive-specific culture cues. While hWJ-MSCs are not classically associated with naive pluripotency induction, their well-characterized secretome—rich in IGF, TGF-β modulators, and other cytokines—may create a paracrine niche conducive to a transitional or “formative-like” pluripotent state [29,30,31]. This partial activation of naive-associated pathways was further enhanced under VALGöX/ALGöX culture conditions. Compared to MEFs feeders, hWJ-MSCs-derived feeders support a more rapid and morphologically distinct conversion to the naive-like state, as evidenced by the formation of dome-shaped colonies and tighter intercellular junctions. These observations suggest that the hWJ-MSCs feeder system not only supports pluripotency but also facilitates epigenetic and morphological transitions characteristic of naive reprogramming.

The feeder-free experiments using hWJ-MSCs-conditioned medium (hWJ-MSCs-CM) further highlight the potency of this paracrine environment. Compared to MEFs-CM, hWJ-MSCs-CM more robustly induced the expression of key naive markers, including *KLF4*, *KLF17*, *ESRRB*, and *DPPA*, alongside significant epigenetic remodeling—namely, reduced H3K9me3 and increased H3K14ac levels. These changes are consistent with chromatin states permissive to naive gene activation and suggest that hWJ-MSCs-CM not only initiates transcriptional reprogramming but also promotes chromatin accessibility in line with naive identity. A notable morphological difference is also observed between the two conditioned media: Rzh11ESCs cultured in hWJ-MSCs-CM exhibited larger cell size relative to those in MEFs-CM. Naive pluripotent cells are known to display a unique metabolic and cytoskeletal profile, including increased mitochondrial activity and cytoplasmic spreading [32,33]. The increased size of hWJ-MSCs-CM cultured cells may thus reflect a shift in metabolic state or cytoskeletal organization associated with naive conversion. We propose that the hWJ-MSCs secretome comprising cytokines, growth factors, extracellular vesicles, and metabolic regulators may modulate key pluripotency-related pathways, including LIF/STAT3, WNT/β-catenin, PI3K/AKT, and TGF-β/SMAD signaling, which are central to pluripotent state transitions and epigenetic remodeling. These observations are consistent with previous studies demonstrating that modulation of LIF/STAT3, WNT/β-catenin, and related signaling pathways plays a central role in naive pluripotency induction and stabilization [32,34]. Our findings highlighted that MSCs-derived extracellular vesicles and exosomes may contribute to chromatin reconfiguration by delivering regulatory RNAs, transcriptional modulators, or epigenetic modifiers that influence histone acetylation and methylation dynamics consistent with the reduced H3K9me3 and increased H3K14ac observed in our study. Additionally, MSCs-secreted metabolic factors may reshape cellular metabolism, which is increasingly recognized as a key determinant of pluripotent state stability and epigenetic plasticity. Notably, although a functional contribution of hWJ-MSCs-derived paracrine signaling to the induction of a naive-associated phenotype has been demonstrated, the specific molecular mediators underlying this effect have yet to be clearly defined. In addition, the present investigation was performed using a single rhesus ESC line, which constitutes a limitation of the study. Even though this cell line is well characterized and extensively utilized in primate pluripotency research, it is well established that pluripotent stem cell lines can display line-to-line variability in transcriptional signatures, epigenetic configurations, and responsiveness to defined culture conditions. Therefore, further studies are warranted to evaluate the robustness and reproducibility of the hWJ-MSCs-derived conditioned medium system across multiple independent rhesus ESC lines, as well as induced pluripotent stem cell (iPSC) lines. Ultimately, such validation would enhance the generalizability and translational relevance of these findings.

Taken together, these results support the conclusion that hWJ-MSCs, through both direct contact and secreted factors, contribute to a permissive microenvironment for the induction and maintenance of a naive-like pluripotent state. While the transition may not be fully complete in all cells, the consistent upregulation of naive-associated transcription factors and accompanying epigenetic modifications point to the activation of a functional naive gene network. Moreover, hWJ-MSCs provide a human-compatible, xeno-free alternative to traditional MEFs-based systems, with important implications for regenerative medicine and interspecies chimerism studies.

## 5. Conclusions

Our findings establish hWJ-MSCs-CM as a xeno-free, human-derived culture system that promotes efficient primed-to-naive conversion under feeder-free conditions, offering improved translational relevance over conventional mouse-based platforms. The generation of a stable naive-like state in non-human primate ESCs holds significant value for regenerative medicine, given the enhanced genomic stability and functional properties associated with naive pluripotency. The use of a clinically compatible conditioned medium further supports the development of safer and more standardized stem cell production strategies. Overall, hWJ-MSCs-CM provides a promising and clinically adaptable platform for advancing naive pluripotency research and its future therapeutic applications. However, a key limitation of this study is that additional functional assays are required to definitively confirm full naive identity and developmental potential.

## Figures and Tables

**Table 1:** List of antibodies used in this study.

Type of Antibodies (Ab)	Antibodies Named	Dilution	Company #Cat. No
Primary Ab	Rabbit anti-OCT4	1:200	Santa Cruz Biotechnology (Dallas, TX, USA) #K0620
	Rabbit anti-NANOG	1:200	Cell Signaling Technology (Danvers, MA, USA) #4903S
	Goat anti-SOX2	1:200	R&D Systems (Minneapolis, MN, USA) #AF2018
	Rabbit anti-TBXT	1:100	Cell Signaling Technology (Danvers, MA, USA) #81694S
	Goat anti-TFAP2C	1:50	R&D Systems (Minneapolis, MN, USA) #AF5059
	Rabbit anti-KLF17	1:200	Sigma-Aldrich (St. Louis, MO, USA) #HPA024629
	Sheep anti-KDM4A	1:50	R&D Systems (Minneapolis, MN, USA) #AF6434
	Rabbit anti-H3K9me3	1:200	Abcam (Cambridge, UK) #ab8898
	Rabbit anti-H3K14ac	1:200	Abcam (Cambridge, UK) #ab52946
Secondary Ab	Goat anti-Rabbit Alexa Fluor 488	1:300	Invitrogen (Carlsbad, CA, USA) #A32731
	Donkey-anti-Goat Alexa Fluor 594	1:300	Thermoscience (Waltham, MA, USA) #A32758
	Goat anti-Mouse Alexa Fluor 568	1:300	Invitrogen (Carlsbad, CA, USA) #A11031
	Goat anti-Mouse Alexa Fluor 488	1:300	Invitrogen (Carlsbad, CA, USA) #M31504

**Table 2:** Primer sequences and annealing temperatures used for qPCR.

Gene Type	Gene	Primer Sequence (5′-3′)	Annealing Temperature (°C)
Housekeeping gene	*GADPH*	F: GGAGCGAGATCCCTCCAAAAT R: GGCTGTTGTCATACTTCTCATGG	54
Pluripotency genes	*OCT4*	F: AGTGTGGTTCTGTAACCGGC R: GACCGAGGAGTACAGTGCAG	58
	*NANOG*	F: AGTCCTGCTTGCAGTTCCAG R: TCAGGTTGCATGTTCGTGGA	55
	*SOX2*	F: AACCAGCGCATGGACAGTTA R: CGAGCTGGTCATGGAGTTGT	56
Primed specific genes	*TBXT*	F: CTTCAGCAAAGTCAAGCTCACC R: TGAACTGGGTCTCAGGGAAGCA	56
	*OTX2*	F: AAAGTGAGACCTGCCAAAAAGA R: TGGACAAGGGATCTGACAGTG	56
Naive specific genes	*KLF4*	F: CCCTACCTCGGAGAGAGACC R: GGATGGGTCAGCGAATTGGA	58
	*KLF17*	F: CCTTACCGCTGCAACTACGA R: ATAGGGCCTCTCACCTGTGT	57
	*TFAP2C*	F: GTTCTCAGAAGAGCCAAGTCG R: TCGGCTTCACAGACATAGGC	56
	*ESRRB*	F: TGCCCTATGACGACAAGCTG R: TGAGCGTCACAAACTCCTCC	58
	*DPPA2*	F: GTACGCCTGCAGTTTCATGC R: TCTATGCCTGGGGATGGGAA	55
	*DPPA5*	F: GGTCGTGGTTTACGGTTCCT R: AGTTTGAGCATCCCTCGCTC	56

## Data Availability

The original contributions presented in this study are included in the article. Further inquiries can be directed to the corresponding author.

## Abbreviations

The following abbreviations are used in this manuscript:

bFGF	Basic fibroblast growth factor
CjESCs	Marmoset ESCs
CyESCs	Cynomolgus monkey
CFU	Colony-forming unit
ESCs	Embryonic stem cells
EpiSCs	Epiblast stem cells
FBS	Fetal bovine serum
FGF2	Fibroblast growth factor 2
NHPs	Non-human primates
HDACs	Histone deacetylases
hWJ-MSCs	Human Wharton’s Jelly-derived mesenchymal stem cells
hWJ-MSCs-CM	hWJ-MSCs-conditioned media
KO-DMEM	Knockout Dulbecco’s modified Eagle’s medium
KOSR	Knockout serum replacement
LIF	Leukemia inhibitory factor
MEFs	Mouse embryonic fibroblasts
MEFs-CM	MEFs-conditioned media
NEAA	Non-essential amino acids
PSCs	Pluripotent stem cells
PSG	Penicillin-streptomycin-glutamine
rhESCs	Rhesus monkey embryonic stem cells
RT	Room temperature
TGF-β3	Transforming growth factor-beta 3
PFA	Paraformaldehyde
SAHH	S-adenosyl homocysteine hydrolase
